# A Dissertation on the Pathology of the Bones, with Illustrative Cases; among Others, a Case of the Removal of Carious Ribs

**Published:** 1828-06

**Authors:** William A. M'Dowell


### PATHOLOGY OF THE BONES.

*A Dissertation on the Pathology of the Bones, with illustrative Cases; among others, a Case of the Removal of carious Ribs.*

By WILLIAM A. M'DOWELL, M.D.

(Continued from p. 412.)

*Internal Necrosis.*—I COME now to speak of a less frequent, but a more deplorable, form of disease.

Necrosis of the internal structure, like external necrosis and most other diseases of the bones, is frequently of constitutional origin. In all such cases, the constitutional taint should first be attended to; then due attention should be devoted to local management, which has always been too much neglected, and to which it is the object of this communication to elicit particular attention.

This disease has its origin in the cancelli, and, from the few cases that have come under my scrutiny, seems frequently to arise from cold and moisture so long endured as thoroughly to *chill*, not only the integuments, but the bones themselves; acting, perhaps, by constriction of their extreme vessels obstructing the capillary circulation, or by causing the metastasis of other diseases to their cancelli.

But internal necrosis, from whatever cause arising, as a natural consequence generates the formation of pus, which generally extends the ravages of the disease through the greater portion of the internal cellular structure of the bones, before the pressure of the accumulated and accumulating volume of matter bursts its way through the external lamellæ; and, until this is effected, and produces distention and pain of the soft and more sensible integuments, the patient is sometimes unaware that he is at all diseased, though, on examination, it is rendered evident that the necrosis had been of long standing.

This is the disease described by BELL and COOPER, under the name of spina ventosa; it is BOYER's internal necrosis; and abscess of the tibia of HEY.

"Spina ventosa, or scrofulous bone," says Bell, "is merely a failure of the internal circulation, a total corruption of the marrow, and a consequent loss of the medullary vessels, by which the whole bone dies, is thrown out by nature, or more frequently the limb must be cut off."

Boyer speaks of internal necrosis as but rarely affecting the articular ends of bones; but as a highly dangerous disease when it does, and generally requiring amputation. Two of the subsequent cases were either of this dangerous kind, or what he distinguishes as caries.

Case.—In the spring of the year 1824, Simon Stair, aged thirteen, of hale constitution, never had been sick; went with a party of boys from a brick-yard, in which they worked, to bathe, and staid long in the cold water; shortly afterwards he complained of headache, and was unable to go on with his work. In a few days the headache ceased, and he was taken with excruciating pain at the right knee, which, with occasional intervals of ease, continued without any swelling or discolouring of the integuments, until some time in the following autumn; when a small abscess, like a common boil, appeared on the anterior surface of the tibia, about an inch and a half below the knee-joint, which soon opened, and formed a sinus. After this he suffered no pain, unless in consequence of fatigue or from exposure to cold. When in attendance on Mr. Stairs' family, in December 1825, I was requested to examine this leg, which I did carelessly and without probing, and informed the boy's father that, from the duration of the disease, I conceived there was carious bone at the bottom of the sinus, but that, from its diminutive extent, the scale might soon be expected to exfoliate, and the disease would get well. Carious portions did frequently separate and pass out, but no amendment followed.

On the 6th of June, 1826, Mr. Stairs again invited my attention to this leg. The sinus appeared much as formerly, but the limb was reduced one half smaller than the other, and was becoming very weak, and he grew more fastidious in his diet.

I now laid the bone bare, and made a particular examination. There was a small round hole through the lamellæ, which discovered a considerable loss of substance within.

I advised an operation to remove the caries, which, with the assistance of Drs. Foot and Caruthers, I performed on the 25th, using a common trepan, and Hey's saw to remove the lamellæ, and detaching the carious cancelli with a gouge and scraper: the caries extended so near the joint that a discharge of synovia followed its detachment, and its continuing to discharge afterwards proved a serious impediment to the healing of the wound. Inflammation, however, being suppressed by blood-letting and the antiphlogistic regimen, the wound healed, and the limb had recovered the full size and strength of its fellow in six months.

Case.—William Camper, a remarkably healthy, active boy, nine years old, much addicted to mischief, often, when engaged in sports, subjecting himself to long endurance of severe cold, was, on the 28th of August, 1826, suddenly attacked with severe pain at the right ankle, and the whole leg speedily swelled, without discolouring the skin.

I visited him on the 1st of September. As he appeared always to have enjoyed remarkably good health until within the last four days, and had no recollection of having ever in any manner injured his leg, I could form no just conception of the nature of the disease. To facilitate the distention of the inflamed parts, and thereby alleviate pain, I took away a few ounces of blood, ordered the application of emollient poultices, and a small dose of salts to be taken every other morning.

Visiting him on the 3d, I discovered the cuticle separated from the cutis vera of the swelled part, by a stratum of albuminous substance. Fluctuation was now very apparent, which seemed nearest the surface about midleg, and extended to the tibia: here I made an opening, and discharged the pus. On the 5th, I made a counter opening near the malleolus internus, passed a seton from one to the other along the denuded bone, and bandaged from the toes to the knee, under the apprehension that the disease was similar to that of Rowland's Tom. This treatment was persisted in until the 22d, when the openings were enlarged sufficiently to admit free examination of the bone; a measure the timidity of my patient had heretofore prevented. I now discovered, to my great surprise, that the disease penetrated, as I imagined, to the interior of the bone, there being a hole, with rugged margins, through the external lamellæ, large enough to admit the end of the finger. I now recommended an operation similar to that I had successfully performed on Stair, with the prognostic that, from the limited duration of the disease, it was necessarily of more limited extent.

With the assistance of Dr. Caruthers, an operation was performed on the 30th. The seton was withdrawn, and the bone laid bare, and brought to view from one sinus to the other. All the lower end of the tibia was diseased; the lamellæ near the articulation with the astragalus constituted but a shell, and might have been crushed together by a strong man between the thumb and fingers; the internal substance was as soft as mortar.

Finding this condition of things, I came to the resolution to remove all the diseased bone, even if it extended to the knee; in which I was cordially supported by Dr. Caruthers.

The anterior surface of the tibia was livid, and emitted no blood when scraped, to about half its length up the bone; the posterior surface exhibited evidence of animated organization more than an inch lower down. To obtain a full examination, interior as well as exterior, I took, with Hey's saw, a wedge-shaped portion out of the anterior surface of the tibia, the apex of which extended to the upper extremity of the livid lamellæ, which discovered the sound lamellæ at this place to consist of a mere shell: necrosis of the cancelli extended considerably higher up the bone. The necrosing cancelli was worked out with a gouge, until blood, evincing vitality, followed the impression of the instrument.

The division of the integuments was then extended, and the bone was divided with Hey's saw an inch higher than it had been penetrated with the gouge. Below, it was divided near the ankle. A small portion of the lower head of the tibia, though diseased, was left to guard against injuring its articulating cartilage.

Thus, about two-thirds of the tibia was removed. The wound was dressed with adhesive straps, over which a roller was loosely applied; due care was taken to prevent unnecessary inflammation, by bleeding, purging, and the antiphlogistic regimen. The small portion of diseased bone left at the ankle was removed by injected washes, and the whole wound healed in about six weeks.

It is very remarkable that this boy could not recollect ever to have felt pain in the diseased leg, until the date first above mentioned, although the extent to which this disease (of notoriously slow progress) had advanced, afforded assurance that it must have been of many months, if not years, duration.

The rapid reproduction of the lost bone was also striking. I less than six months he was able to walk without crutch or stick, and could not be prevented. At this time (August 12th, 1827,) he conceives the leg as stout as it ever was.

At the posterior superior end of the removed portion of bone were several clusters of callus, discovering the tendency to reproduction, whilst the devastation was yet progressing.

The present deformity of this leg consists in but little else than the scar. The new bone, to the touch, seems about a fourth larger than that of the other leg, and much rougher.

Case.—Betsy Gray, a young woman of scrofulous diathesis, at the age of eighteen, when in delicate health, was, in March 1823, exposed a length of time to inclement weather.

About three weeks after, she experienced frequent pain of the right foot, and was perpetually troubled with uneasy sensations of the foot and leg: the foot regularly swelled during the day, and subsided at night. Such was her case, without much variation, until May, when pain became more intense, the swelling increased, and a sinus formed near the anterior articulation of the os cuboides.

In the spring of 1824, an abscess appeared on the right side of the chest, about the seventh rib, which her attending physician endeavoured to discuss: pus, however, effected its discharge in the following September, first, by a sinus situated about four inches from the anterior end of the rib, and, some weeks after, by another situated at the angle.

Such is the account given by Miss Gray of the early stages of her disease, which continued with but little variation, under different plans of treatment, pursued by several physicians, until March 1827, when she was brought to town, and came under my care. There were at this time five issues from the foot, two from the os calcis, one from the cuboides, and two from the ossa cuneiforme, all communicating with one another: fluids injected into any one of them issued from the other. Through none of those openings was I enabled to examine the condition of the astragalus: all their courses tending below it suggested the hope that it was sound.

As, from Miss Gray's own account of her case, the disease of the foot was ten or twelve months prior to that of the side, I conceived the latter might owe its origin and its maintenance, at least in part, to sympathy with the former; and my recommendation of removal of the carious bones of the foot was predicated on the hope that removal of this source of excitation might produce such a change in the side as would cause the rib to exfoliate or resume healthy action, as in Miss Harvey's case. As there were no symptoms indicating disease of the leg bones, and as there were grounds for hope that the astragalus might be sound, in preference to amputation, removal only of the diseased bones of the foot was advised; well convinced, from my experience of the power of nature in such cases, that bones would be speedily reproduced, or such other substance as would better supply the deficiency than any mechanical contrivance we could substitute for a leg.

April 3d.—With the assistance of Drs. Carruthers, Shanks, Weir, and Preston, all the tarsal bones were removed: they were so completely carious and soft, that they were easily crushed between the thumb and fingers, the astragalus not excepted. No part of their structure retained the natural appearance, but their cartilaginous articulating surfaces. On their removal, the bones of the leg were found so much diseased that the finger met but little resistance in passing its full length up into the tibia; whereupon, with as little delay as practicable, the leg was amputated below the knee: but the disease was not yet circumvented, remaining portions of both bones were thoroughly carious. It was now concluded to remove the remaining portions of the tibia and fibula, and examine the os femoris, to ascertain whether that bone was sound. Finding that it was so, and my patient being much exhausted, I trimmed off a considerable portion of cartilaginous surface from its articular condyles, and, neatly adjusting the ample flap I possessed, I dressed the stump.

No uncommon symptoms ensued; as much of the wound as usual healed by the first intention; the occasional discharge of synovia probably retarded the subsequent healing process; but in six weeks it was completely cicatrised.

*Removal of carious Ribs.*—Miss Gray's side now became an object of interesting attention: it remained in statu quo. Hectic fever, under which she had already laboured when she first came under my care, was excited and increased by the hot weather; colliquative diarrhœa came on; the discharge from her side, always extremely fetid, grew more so; her strength declined daily.

From many careful examinations, I became satisfied that the caries of one or more of the ribs was to the extent of six or seven inches. From the anterior to the posterior opening, injected fluids readily passed; but not vice versa. The lungs were involved in the disease, as was evident from a troublesome and alarming cough, with purulent expectoration, with which she was, and long had been, much harassed; and which caused me to remark to her that the removal of those ribs would afford a prospect of its relief. She caught at the prospect with avidity, and urged the operation, which she was much encouraged in by the concurrent opinions of other physicians who visited her.

Fearful of the result, her case being a very unfavorable one to success, I for some time parried her solicitations. I very much doubted whether the existing disease in the lungs might not be too extensive to admit of cure even after the caries, which I considered the proximate cause, was removed. Finally, however, I consented to operate.

Never having seen a detailed mode of operating for the removal of a rib, I made my first essay upon a dog, and found the operation less difficult than I had anticipated.

On the 25th of June, 1827, with the advice and assistance of Drs. Caruthers, Shanks, and Jordan, the operation was performed.

An incision was made upon the seventh rib, from the posterior to the anterior sinus, bringing to view about six inches of its convex surface, discovering it to be in a state of complete necrosis. No formation of callus anywhere discoverable. After examination, the incision was carried forward about an inch and a half farther, where the rib appeared sound, bleeding when scraped. Having completely divested it of its periosteum, I here passed an elevator under the rib and between it and its periosteum, and divided it with Hey's saw. The incision was then extended back to the spine, and the rib was there detached from its articulation with the vertebræ. At this part of the operation much care was used to avoid wounding the dorsal nerves.

On farther examination, the sixth rib was found in like manner diseased, and was in like manner removed. Underneath this rib, a little anterior to the angle, a sinus communicating with the lungs was discovered: a hole about the size of a goose's quill, which emitted a great quantity of matter, discharging with more freedom when she coughed.

From reviews I have seen of operations to remove carious ribs, as performed by Cettadini and Richerand, securing the intercostal arteries seems to have constituted a difficulty.

In this operation I was not under the necessity of applying a ligature to any blood-vessel; the intercostals lying between the pleura and the periosteum were made perfectly secure by interposing the elevator, as above stated, between the periosteum and the rib.

After removing as much of the diseased soft parts as was deemed prudent, the wound was closed with the interrupted suture and adhesive strips, covered with lint, and secured with a roller. Inordinate inflammation was completely prevented by two small bleedings, and maintaining a loose state of the bowels.

Examination on the fourth day discovered about four-fifths of the wound to have healed by the first intention. No pain has been complained of since that time; the hectic declined, and disappeared in a few days; the cough has materially subsided, sometimes not troubling her for twenty-four hours together. An issue is maintained by tents, opposite the sinus of the lungs, through which a considerable quantity of matter, which appears to be a mixture of mucus and pus, discharges: this opening will be maintained until the lungs are entirely relieved.

On the 15th August, she left town on a visit to Digu's sulphur spring, a trip of twenty-two miles, which she made in a rough Jersey waggon, without complaining of fatigue. I visited her on the 22d: her cough had mended, her appetite and strength had increased, and her general health and appearance had visibly improved. She rides daily to the spring on horseback, a distance of more than half a mile from her lodgings.

The portions of rib removed had suffered internal necrosis. Their lamellated structure was to a small extent destroyed at several points: viz. at the anterior sinus, at the angle, and at the neck; but most at the angle.

The event of the preceding cases, together with others, affections of bones and joints, not here detailed, treated upon the same general plan, with results equally satisfactory, have induced me to adopt the conclusions, that *dead* bones should be considered and treated as foreign bodies; that they are as foreign to the living bone, or parts with which they are in contact, as though they had been introduced from without; and that they should be removed thence without delay, even though it were an entire bone, for, the greater the oppression, the greater the necessity for relief.

Were a surgeon called to a patient who had stuck a snag of wood, or (to render the analogy more palpable) say a sharp piece of bone, deep into his flesh, what would we think of his awaiting its removal by the efforts of nature? Yet we know that, after a time, (if in the interim the patient should not die,) her process would effect its expulsion. Inflammation would expand and relax the surrounding parts, which would presently terminate in mortification or suppuration: if the latter, the outlet would be enlarged and lubricated by the discharge, and the snag, after a tedious interval, would be forced out by the pressure of granulations formed within.

Between the cases of a carious plate under the periosteum and the snag in the flesh, I conceive a strong analogy to exist, though differing in the following particulars: the former is in contact with parts less sensible, and endued with less active vitality; consequently the processes of inflammation and relaxation, with their necessary consequences, mortification or suppuration, or both, are of slower progress, less painful, and more insidious.

The snag, having no obstacle to its exit except from superincumbent integuments, is more easily pressed out by the granulations underneath, than the carious plate, which, together with the same obstruction, is confined by the connecting processes of the lamellæ, rendering force requisite to detach it, even after the integuments are removed.

Upon the whole, I can conceive no other reason for preference of delay in the one case to the other, except that in the case of caries the progress of disease is slower, more sufferable, and less immediately threatening to life. Nature will frequently accomplish relief in both cases; but oftener, I believe, and with more facility, from the snag than from the caries. But why in either case await for months a result that a little skill would, with less hazard, effect in a moment?*

---

### HYDROPHOBIA.

* The American Medical Recorder, No. xli.

